# A Systematic Review of Secondary Traumatic Stress in School Personnel: A Synthesis of Quantitative Findings

**DOI:** 10.1111/josh.70087

**Published:** 2025-09-15

**Authors:** Paige M. Klemme, Barbara Pierce, Jack E. Turman, James R. Brown, Sadaaki Fukui

**Affiliations:** ^1^ Indiana University School of Social Work Indianapolis Indiana USA

**Keywords:** Professional Quality of Life Scale (ProQOL), school personnel, secondary traumatic stress (STS), Secondary Traumatic Stress Scale (STSS)

## Abstract

**Background:**

Secondary traumatic stress (STS) in school personnel is under‐researched, with limited data on its frequency and susceptibility. This systematic review examines the extent of STS and the factors contributing to it among school personnel, defined as individuals employed or contracted by US school systems.

**Methods:**

The review includes only studies that utilized the Professional Quality of Life Scale (ProQOL) and/or the STS Scale (STSS). A comprehensive search identified 18 peer‐reviewed publications (2012–2021).

**Findings:**

Thirteen authors used the ProQOL, while six used the STSS, with half of the latter reporting moderate or higher STS levels. Studies using ProQOL scoring methods found average STS levels. Limitations include a lack of sample diversity, as most participants were white (32.4%–97.2%), female (70.9%–93.2%), and teachers (12/18 studies). Many variables had inconsistent findings across studies. Other variables such as leadership practices and school safety showed significant associations with STS.

**Implications:**

These data can be used to better understand factors associated with STS and to inform the development of effective preventative and reactive strategies to reduce the impact of STS on school personnel.

**Conclusion:**

Continued research needs to occur assessing STS in school personnel to better inform best practices for prevention and reactive strategies.

## 1 Introduction

Secondary traumatic stress (STS) refers to the symptoms of post‐traumatic stress disorder (PTSD) that result from second‐hand exposure to trauma experienced by others, and school personnel, defined as individuals employed or contracted by US (k‐12) school systems, are at risk for secondary exposure to a traumatic event [1]. School personnel are at an increased risk of being exposed to second‐hand accounts of trauma. More than two thirds of children in the United States reported at least one traumatic event by the age of 16 [2]. School personnel are exposed to children who have experienced adversity and are the most common reporters of abuse and neglect, with 19.4% of the allegations [3]. The exposure to students experiencing adversity puts school personnel at risk for STS.

The onset of STS symptoms can be sudden, including intrusive thoughts, avoidance behaviors, negative cognitions, and hyperarousal. However, unlike PTSD, STS symptoms occur due to secondary exposure, not direct, primary exposure to trauma [1]. These symptoms can impact school personnel professionally and personally. STS has been shown to decrease job effectiveness, burnout, and impacts professional relationships, and cause psychological and physical health issues [1, 4, 5, 6]. There is a need for a comprehensive understanding of STS in school personnel to decrease such negative outcomes by informing future studies and strategies to prevent STS in personnel and identify practices to support those affected by STS.

STS has been studied across disciplines and occupations including child welfare workers, mental health providers, nurses, and social workers [5, 7, 8, 9]. There has been a limited focus of STS in school personnel. The term compassion fatigue (CF) tends to be more readily studied in school personnel, especially teachers [10].

A recent systematic review focused on CF in teachers, which addressed some of the same studies presented in this article [10]. However, even though some of the same studies are used, this systematic review focuses on STS in school personnel and not CF in teachers. When STS was initially coined, STS was believed to have occurred due to the empathy the professional felt for the patient, which caused a stress response resulting in STS symptoms; thus, CF and STS were used interchangeably [1]. Over the years, STS has been viewed as a separate concept from STS as CF is considered a more chronic, broader, multi‐dimensional term, whereas STS can be acute in nature with a specific focus on symptoms from a secondary exposure [11, 12, 13]. For the purposes of this paper, STS and CF are viewed as two separate constructs. In an effort to provide clarity on the frequency of STS, this systematic review will focus on the two most common quantitative measures found in the literature assessing STS: the Professional Quality of Life Scale (ProQOL) and the Secondary Traumatic Stress Scale (STSS) [10, 13].

This systematic review synthesizes the existing quantitative literature regarding STS in school personnel by reviewing existing knowledge, highlighting gaps, and identifying contradictory findings with a focus on looking at articles that used the ProQOL and/or the STSS. The research questions to be addressed in this systematic review include: (1) What is the pervasiveness of STS in school personnel? (2) What factors are associated with STS in school personnel? The information synthesized from this systematic review can help better understand the extent of STS among school personnel and inform both preventive and reactive measures to mitigate its impact.

## 2 Methods

This systematic review follows the preferred reporting items for systematic reviews and meta‐analyses (PRISMA) checklist with Joanna Briggs Institute (JBI) critical appraisal for cross‐sectional studies [14, 15]. No systematic review focusing on school personnel and STS has occurred prior to December 2024. This systematic review focuses on school personnel, recognizing that various roles in schools experience secondary exposure to trauma, distinguishing STS from CF. It targeted quantitative studies on STS in school personnel. Inclusion and exclusion criteria were established before the initial searches.

The target studies had to include school personnel as participants, defined as anyone employed by or contracted through a school system serving grades K‐12 in the United States. The studies also needed to provide quantitative STS results. This systematic review excluded studies with a focus on solely CF, vicarious trauma, and/or burnout as they are viewed as separate concepts. Qualitative studies, perspective articles, gray literature, unpublished papers, and conference abstracts were excluded, along with studies conducted outside of the United States.

**FIGURE 1:**
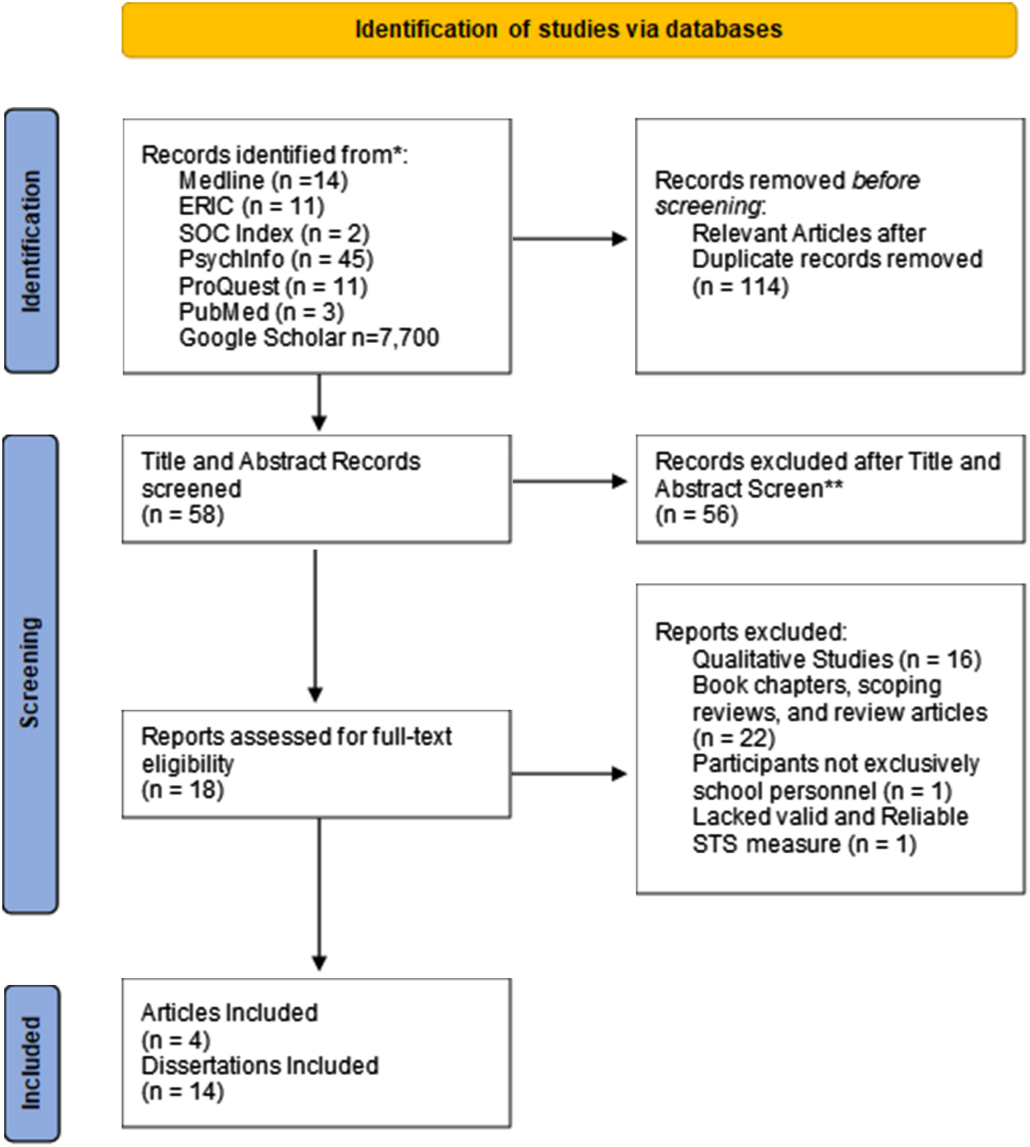
Secondary traumatic stress in school personnel systematic review PRISMA flow diagram. (Chart adapted from Page et al. 2021).

The search strategy followed the PRISMA structure (see Figure 1). The study search and extraction were conducted between December 2021 and January 2022. The primary database search was EBSCO with Medline, ERIC, PsycINFO, and Socindex, followed by ProQuest, PubMed, and Google Scholar. The key words were the same for each of the search terms “ab (school personnel OR teachers) AND ab (secondary trauma OR STS).” The time frame for the articles was unspecified. The keywords were selected after several preliminary searches to identify relevant terms. Articles mentioning school personnel (K‐12) and secondary trauma in the abstract and/or title were recorded in the initial search. Duplicates within the same database were excluded, but duplicates from different databases were initially included. The initial search in Google Scholar was concluded when there were no relevant articles in the prior 25 articles listed. Articles that found on Google Scholar were checked to ensure they were peer reviewed.

The data collection included title and article screening, followed by full text screening. During the full‐text screening, 10% of articles were assessed by a second reviewer. While data are recommended to be extracted by a minimum of two people, it is acceptable to have one person extract data with a second person to check for accuracy and completeness [16].

The studies in this systematic review included 17 cross‐sectional design studies and one longitudinal study. The JBI critical appraisal checklist for analytical cross‐sectional studies was used as a quality appraisal assessment [15]. One study was longitudinal and does not follow the cross‐sectional design, which is a limitation in using this form of evaluation. Preparation of this paper did not involve primary research or data collection involving human subjects and therefore, no institutional review board examination or approval was required.

## 3 Findings

The initial search returned 114 relevant results. After the title/abstract screening, 56 articles were excluded, leaving 58 that met the criteria. During full‐text screening, 40 articles were excluded: 16 focused solely on qualitative methods, and 22 were book chapters, scoping reviews, or review articles. Two more articles were excluded—one was about an adjacent topic but did not report on school personnel, and the other used an unvalidated self‐created STS measure. The full‐text screen yielded 18 relevant results. Following the screenings, 18 studies met the inclusion criteria and utilized the ProQOL or STSS.

The 18 studies ranged from 6 to 450 participants (*N* = 2583 when combined). Seventeen of the studies were cross‐sectional designs, and one was a longitudinal design. The locations of surveys included Midwest [17, 18, 19], West [20, 21, 22, 23], Northeast [24, 25], Southeast [26, 27, 28], and nationally [29, 30, 31]. Three articles did not provide a specific region within the United States [32, 33, 34].

Most participants in each study were female, ranging from 70.9% to 93.2%. Other descriptive information varied across studies, with some not providing descriptors or categorizing them differently. In studies that treated age as a continuous variable, the means ranged from 39.9 to 45.6 years. White participants made up the majority of the sample, ranging from 32.4% to 97.2% in the 12 studies that reported race. One study had a sample of Asian Americans (34.6%) which surpassed the white counterparts (32.4%), and had Latinx participants (24.3%) [23]. Studies that had white participants as the majority but had a second race making up over 10% of the sample include: 20% of the sample identifying as Native American [20], 15.3% of Latinx participants [22], 10.2% Black participants [26], 32.4% Black participants [27], 15.3% Black and 12% Latinx participants [31], 15.2% Hispanic participants [34], and 16.7% Black participants [18].

Most of the studies solely focused on teachers [17, 20, 21, 22, 23, 25, 26, 27, 28, 29, 30, 32]. Two focused on school counselors or social workers [19, 33], and four explored various forms of school personnel [18, 20, 24, 34].

There were multiple scales used to measure STS or aspects related to STS. For the purposes of clarity, the results were assorted based on their measure of STS. The measures included in this systematic review focus on the ProQOL and the STSS. The ProQOL was derived to assess for CF and comprises three subcategories: compassion satisfaction, burnout, and STS [35]. This systematic review will only focus on the STS subcategory (10‐items, five‐point Likert scale) results. The STSS is a 17‐item, five‐point Likert scale with three subcategories including intrusion, avoidance, and arousal [36]. Most of the studies used the ProQOL or the STSS; however, two studies used both measures [20, 23].

### 3.1 R1: How Pervasive Is STS in School Personnel?

The STS means from the corresponding articles are reported to better understand the prevalence of STS in schools. Tables 1 and 2 display the STS means gathered from the ProQOL. Table 1 focuses solely on the ProQOL means from the STS subscale. Nine out of 16 studies used this method to assess STS. A subscale score below 22 indicates low STS, 23–41 indicates average STS, and 42 or more indicates high STS [35]. All nine articles had overall means within the “average STS level.”

**TABLE 1:** ProQOL STS subscale results.

Author(s)	*N*	STS mean	STS SD	STS level
Borntrager et al. (2012)	241	23.00	6.10	Average
Christian‐Brandt et al. (2020)	163	23.15	6.91	Average
Gomez (2021)	65	24.09	5.48	Average
Hydon (2016)	136	23.04	7.94	Average
Rankin (2021), Female	120	27.04	5.87	Average
Rankin (2021), Male	38	22.00	5.49	Low
Santa (2017)	58	28.40	2.94	Average
Shoieb (2020)	132	22.47	5.08	Average
Vanderwill (2021)	23	22.00	7.84	Average
Wilson (2020)	89	22.99	5.75	Average
Total	1065			

**TABLE 2:** ProQOL STS composite score results.

Author(s)	*N*	STS mean	STS SD	STS level
Grybush (2021)	147	52.37	0.58	Average
Rumsey (2017)	174	49.61	9.49	Average
Simon (2020)	88	50.00	10.00	Average
Steen (2020), general education	110	50.96	9.90	Average
Steen (2020), special education	60	49.40	10.05	Average
Total	579			

The ProQOL includes three subcategories: compassion satisfaction, burnout, and STS. An alternative method for scoring STS in the ProQOL is to include the other two subscales (compassion satisfaction and burnout) [35]. Previously, a study demonstrated the unidimensionality of the construct, recommending the use of a single score rather than separate subscales [37]. Therefore, the ProQOL scores listed in Table 2 include the sum of all subcategories. Four studies used this method to report STS levels. Scores between 44 and 56 indicate an “average STS level” [35], and all four studies had a mean score within the average STS level range.

Six studies used the STSS to determine the mean STS. The scoring for the STSS is as follows: little or no STS (< 27), mild STS (28–37), *M* = moderate STS (38–43), high STS (44–48), and severe STS (49+) [36]. Table 3 presents the mean scores from the STSS. Three studies had mild or little STS [23, 29, 34]. Two studies had moderate levels of STS, and one study had a severe level of STS.

**TABLE 3:** STSS mean scores.

Author(s)	*N*	STS mean	STS SD	STS level
Borntrager et al. (2012)	241	39.00	13.70	Moderate
Branson (2021)	6	51.00	12.21	Severe
Denham (2019), blighted	88	34.03	13.98	Mild
Denham (2019), not blighted	84	20.80	13.38	Little or no
Hydon (2016)	136	28.78	5.11	Mild
Steketee (2020)	450	42.06	14.79	Moderate
Stevens et al. (2020)	167	32.47	11.90	Mild
Total	1172			

The three STS mean scoring methods included the ProQOL STS subscore, ProQOL composite score, and the STSS score. Both forms of ProQOL scoring (subscore and composite) found participants to have average levels of STS. Thirteen of the authors used the ProQOL, and six authors used the STSS. Half of the authors who used the STSS had mean scores of moderate or higher levels of STS.

Some studies only presented frequencies and did not complete further quantitative analysis. These studies used mixed methods, and their findings and factors were qualitative and are excluded from the following section of the systematic review [21, 24, 31].

### 3.2 R2: What Factors Increase or Decrease STS in School Personnel?

This section focuses on factors that may increase or decrease STS. This section provides a brief description of the relevant studies and their findings along with the statistical analysis used. The compiled results of factors can be found in Table 4. This section describes factors that were analyzed by the corresponding authors, focusing on statistically significant results.

**TABLE 4:** Secondary traumatic stress factors.

Variables	Statistically significant	Not statistically significant
Gender	(Rankin 2021; Shoieb 2020)	
Trauma/stress/hazards	(Rankin 2022; Rumsey 2017; Simon 2020; Steen 2019; Stevens et al. 2020)	(Borntrager et al. 2012; Grybush 2021)
Years' experience		(Gomez 2021; Shoieb 2020; Rankin 2022)
Teacher/school type	(Denham 2019; Gomez 2021; Shoieb 2020)	(Steen 2020; Gomez 2021)
Seeking other employment	(Borntrager et al. 2012)	(Christian‐Brandt et al. 2019)
Burnout	(Anama‐Green 2020; Hydon 2016; Steen 2019; Grybush 2021; Rumsey 2017)	(Hydon 2016)
Interventions: Self‐care/mindfulness/professional development	(Anama‐Green 2020; Vanderwill 2021) (Sept. and June)	(Grybush 2021; Vanderwill 2021) (Dec. and Feb.)
Self‐efficacy/empathy/cognitive reappraisal	(Rumsey 2017; Simon 2020)	(Rumsey 2017)
Compassion satisfaction	(Grybush 2021; Hydon 2016; Steen 2019)	(Hydon 2016)
Leadership practices and school safety	(Borntraeger et al. 2012; Wilson 2020)	

Shoieb found statistical significance in STS (ProQOL, *N* = 132) between female and male participants, where female indicated higher STS [25]. Rankin found (ProQOL, *N* = 158) statistical differences in STS between females (*N* = 120) and males (*N* = 38) with females indicating higher levels of STS [30].

Stevens et al. studied school shooting media exposure and STS (STSS, *N* = 167) in school personnel [34]. STS was found to be positively correlated weakly with verbal aggression toward teachers and indirect aggression toward teachers. Rumsey found STS (ProQOL) to have a weak correlation with secondary exposure to childhood trauma [33]. The secondary exposure to childhood trauma measure analyzes the frequency (Likert scale 1–7) of 13 listed secondary exposures. All participants indicated secondary exposure; this measure was simply looking at the frequency of exposure.

Simon found STS (ProQOL, *N* = 88) was associated with increased student socio‐emotional difficulties and teacher Adverse Childhood Experiences survey (ACEs) [27]. There was statistical significance between teachers who had a history of trauma and those who did not in the t‐test analysis [30].

Shoieb [25] found elementary teachers had statistically higher STS scores than middle school teachers (ProQOL, *N* = 132) [25]. Gomez used a *t*‐test to assess differences in STS (ProQOL) and teacher type [32]. General education teachers (*N* = 33) were found to have statistically significant lower rates of STS than all the other teachers (*N* = 32). General education teachers were found to be in the low category and all other teachers were within the average category.

Denham used a t‐test to assess STS (STSS) differences between blighted (*n* = 88) and non‐blighted schools (*n* = 84) [29]. Blight was measured by the School Disrepair Index, and on average, personnel in blighted schools experienced mild symptoms of STS while personnel in non‐blighted schools experienced little to no STS.

Borntrager et al. found STS (STSS) was weakly correlated with seeking other employment (A similar result was found using the ProQOL) [20]. Borntrager et al. used a multiple regression model (*N* = 233) which found seeking other employment and employers encouraging talking with peers about stress accounted for 13.3% of the variance of STS (using the STSS). A similar model using the ProQOL with STS as a dependent variable showed that the same two independent variables accounted for 9% of the variance [20].

Anama‐Green reported that STS (ProQOL) was moderately correlated with burnout (*N* = 144) [17]. Hydon found that there was a significant moderate correlation between STS (ProQOL, *N* = 136) and burnout and a weak negative correlation between STS and compassion satisfaction [23]. Steen found that STS (ProQOL, *N* = 260) was significantly moderately correlated with burnout, weakly correlated with professional distress, and negatively weakly correlated with compassion satisfaction [28]. Grybush found that STS was moderately correlated with burnout and was negatively correlated with compassion satisfaction [26]. Rumsey found STS had a moderate correlation with burnout [33].

There were a handful of studies that looked at the association between STS and various interventions such as mindfulness, professional development, and self‐care. Anama‐Green reported that STS (ProQOL) was negatively moderately correlated with intrapersonal mindfulness [17]. Vanderwill used a longitudinal design to measure self‐care's association with STS (ProQOL, *N* = 27) among four time points in the year September, December, February, and June; however, all of which were indicative of low STS [18]. There was a negative moderate correlation between STS and self‐care in September (*N* = 25) and June (*N* = 23). There was no statistical significance for December and February.

Rumsey found STS (ProQOL) in school counselors (*N* = 174) using convenience sampling had a negative correlation with secondary trauma self‐efficacy [33]. A hierarchical regression was completed. The first step explored self‐efficacy and was statistically significant, accounting for 34% of the variance. The second step added empathy to the model, and the model became statistically insignificant. Cognitive reappraisal was found to be negatively associated with STS [27].

Borntrager et al. found STS (STSS) was weakly negatively correlated with employers encouraging personnel to talk about stress with peers [20]. Wilson explored organizational factors and STS (ProQOL, *N* = 89) and found the domains of promoting physical and psychological safety (accounted for 34% of variance) and STS informed leadership practices (28% of variance) were statistically significant [19]. These were separate regression models. While there were many variables assessed for an association with STS, there seemed to be some inconsistencies. The next section will provide additional context as to why there may be so much variability in results, common limitations, overall limitations of this systematic review, and next steps.

## 4 Discussion

This systematic review found that while the average levels of STS were consistent, but variables associated with STS often varied. The STS reported from the studies using the ProQOL were the most consistent, with the average participant in the studies indicating an “average level of STS.” In contrast, half of the studies that used the STSS indicated a moderate or higher level of STS. A moderate score or above on the STSS indicates that the participant meets criteria for PTSD [13]. While the STSS is not a diagnostic tool, it does indicate that further mental health assessment could be appropriate due to moderate or higher levels of STS.

A limitation in comparing the results of the systematic review is not knowing the overall distribution within the categories of the included studies in the systematic review. For instance, the mean provides an overall indicator of the average STS, but it is unclear how many score in the “high” category for the ProQOL and “moderate” or higher for the STSS. The use of two different measures makes it difficult to grasp the prevalence of STS.

Bontrager et al. and Hydon used the ProQOL and the STSS; interestingly, Bontrager et al. had average levels of STS from the ProQOL but moderate levels of STS from the STSS, whereas Hydon had average levels of STS from the ProQOL and mild STS from the STSS [20, 23]. This may indicate that the STSS has more sensitivity in discerning levels of STS, as evidenced by the STSS having mild or moderate average designations while the ProQOL had an average designation for both studies. It is important to distinguish between different levels of STS, especially if the goal is to determine if additional support is needed.

In addition to the categorization and different measures, another limitation is the lack of generalizability as none of the studies used random sampling. The main sampling methods for the systematic review were convenience, followed by snowball, and then purposive. It should be noted that the scores from the ProQOL and STSS are not representative of the schools from which the samples are drawn and are not representative of school personnel in general. Further studies are needed.

Overall, it seems that risk factors impacting STS differed; this could be due to the individuality of each school system. The factors assessed in this systematic review are split into personal, work factors, and school. Personal factors include factors the person experienced outside of their working experience (ex. Gender and trauma history). Noticeably, there is a knowledge gap when it comes to race. White participants made up the majority of the sample, ranging from 32.4% to 97.2% in the twelve studies that reported race. In one study, Asian Americans were the majority of the sample [23]. There were studies that had white participants as the majority but had a second race making up over 10% of the sample [18, 20, 22, 26, 27, 31, 34]. It is unclear how STS translates when it comes to non‐white races. To address this gap, future studies should focus on sampling or over‐sampling more diverse areas.

Most of the participants were female, and two studies assessed STS gender differences. Females were found to have higher levels of STS than their male counterparts [25, 30]. Having a trauma history had more inconclusive results. There are a few potential reasons for this ambiguity around the history of trauma. One is that different measures were used to assess trauma. Two studies used ACEs [26], one used a three items questionnaire [20], and another asked a dichotomous question about the history of trauma [30]. Another reason is that two of the studies only looked at childhood adversity rather than a lifetime history of trauma. While a history of trauma was found to be related to higher levels of STS in two studies from the systematic review, future studies need to occur where a consistent measure is used to operationalize trauma history.

Work factors were another domain identified in the systematic review. Work factors included years of experience, teacher type, and compounding exposures. The studies that assessed experience found that years of experience were not significantly related to STS levels [25, 30, 32]. Each study split experience into the novice or non‐tenured teachers (roughly less than 5 years of experience) and veteran or tenured teachers (greater than roughly 5 years). A potential reason that years of experience was not statistically significant was due to this dichotomous grouping. Another reason this may occur is due to the idea that the personnel most impacted by STS seek other employment opportunities. So those who experience STS to the point of distress seek other employment to alleviate the distress. Borntrager et al. [20], found that there was a weak correlation between seeking other employment and STS as well as found it significant in the overall regression model. Future studies may want to explore turnover and STS as burnout was found to be correlated with STS when using the ProQOL [17, 23, 26].

School factors assessed in STS school personnel literature include professional development, employers encouraging personnel to discuss stress with peers/STS leadership practices, school safety, and trauma‐informed care. One study looked at professional development and found that it was not significantly associated with STS [26]. Another school‐level protective factor assessed in the literature had to do with leadership. Bontrager et al. found a weak negative correlation between employers encouraging personnel to talk about stress with peers, and the same article also found that this was statistically significant in the regression model [20]. Similarly, Wilson [19] found that STS‐informed leadership practices accounted for 28% of the variance for STS. This provides hope that leadership within schools can help insulate their personnel by providing supportive proactive strategies such as facilitating supportive discussion with peers. Additionally, Wilson found that physical and psychological safety accounted for 34% of the variance of STS [19]. Thus, a sense of safety accounted for STS, which makes sense since STS is a reaction to a perceived threat from a secondary source. An unsafe space in addition to a secondary exposure of trauma could compound one another.

Means were used to address the first research question. This decision was made based on the common descriptive statistics reported in the studies. From the mean, this systematic review was unable to clearly report how many are impacted by high levels of STS (ProQOL) or moderate and higher levels of STS (STSS). Further research needs to be completed on school personnel to determine the number impacted by moderate to high STS levels.

With so few studies conducted measuring STS in school personnel, a lack of consistent operationalization of variables, and a lack of generalizability, there is a need to replicate studies that have already been conducted. The variation in the sample is a limitation. School personnel were chosen as the focus for this systematic review as many roles within a school are potentially impacted by STS; however, many of the studies focused on teachers. This systematic review may not provide an accurate generalization for all school personnel.

Many of STS recommendations from the studies focus on the individual or the school interventions; however, they fail to consider what would be preventive versus what would be best reactive strategies to help those with moderate or higher levels of STS. Currently, there is no study that looks at formal interventions addressing STS in school personnel. Future studies should assess efficacy, or these recommended interventions and literature should begin to be gathered about evidence‐based practices for school personnel and STS using a standardized tool to assess STS.

STS can manifest in school personnel, yet there are many gaps in the current literature. The issue of STS needs to be further explored in school personnel, and evidence‐based practices and interventions need to be developed to prevent or address STS. Due to the lack of studies on STS and school personnel, fields with similar levels of STS and developed evidence‐based interventions may be useful to combat STS in school personnel.

## Conflicts of Interest

The authors declare no conflicts of interest.
